# Capturing RNA–protein interaction via CRUIS

**DOI:** 10.1093/nar/gkaa143

**Published:** 2020-03-06

**Authors:** Ziheng Zhang, Weiping Sun, Tiezhu Shi, Pengfei Lu, Min Zhuang, Ji-Long Liu

**Affiliations:** 1 School of Life Science and Technology, ShanghaiTech University, Shanghai, China; 2 University of Chinese Academy of Sciences, Beijing, China; 3 Shanghai Institute of Biochemistry and Cell Biology, Chinese Academy of Sciences, Shanghai, China

## Abstract

No RNA is completely naked from birth to death. RNAs function with and are regulated by a range of proteins that bind to them. Therefore, the development of innovative methods for studying RNA–protein interactions is very important. Here, we developed a new tool, the CRISPR-based RNA-United Interacting System (CRUIS), which captures RNA–protein interactions in living cells by combining the power of CRISPR and PUP-IT, a novel proximity targeting system. In CRUIS, dCas13a is used as a tracker to target specific RNAs, while proximity enzyme PafA is fused to dCas13a to label the surrounding RNA-binding proteins, which are then identified by mass spectrometry. To identify the efficiency of CRUIS, we employed NORAD (Noncoding RNA activated by DNA damage) as a target, and the results show that a similar interactome profile of NORAD can be obtained as by using CLIP (crosslinking and immunoprecipitation)-based methods. Importantly, several novel NORAD RNA-binding proteins were also identified by CRUIS. The use of CRUIS facilitates the study of RNA–protein interactions in their natural environment, and provides new insights into RNA biology.

## INTRODUCTION

RNA-binding proteins (RBPs) play important roles in various biological processes such as regulation, splicing, modification, localization, translation and stabilization of RNAs. Many RBPs, including some proteins that lack the classical RNA-binding domains, have distinct spatial and temporal distributions in cells and tissues. The malfunction of RBPs is responsible for many human diseases (1–3). In order to gain insight into the function of RBPs, it is necessary to identify detailed interactions between the RNA and its binding proteins. In general, studying the interaction between proteins and RNAs includes two classes of methods: the protein-centric and the RNA-centric methods.

The protein-centric class involves all immunoprecipitation-based methods, targeting to a protein of interest and analyzing the bound RNAs. Initially, the RNA immunoprecipitation (RIP) assay, which was adapted from the chromatin immunoprecipitation assay (ChIP) (4), was used to identify RNA–protein interactions. However, because the RIP assay retains protein-protein interactions, it is not well suited for studying direct RNA–protein contacts. To exploit zero-length covalent RNA–protein cross-linking and RNA fragmentation, a method named crosslinking and immunoprecipitation (CLIP) has been developed (5). This involves directly illuminating cells or tissues with UV-B light, which catalyzes the formation of covalent bonds between RNA and proteins that are in direct contact. Later, photoactivatable-ribonucleoside-enhanced crosslinking and immunoprecipitation (PAR-CLIP) was developed to further improve the cross-linking efficiency of CLIP (6).

The RNA-centric class is oligo-capture based approach, which targets to a given RNA and captures its bounding proteins. The examples include RNA antisense purification-mass spectrometry (RAP-MS) and comprehensive identification of RNA-binding proteins by mass spectrometry (ChIRP-MS) (7,8), both of which use biotin-labeled DNA fragments complementary to the target RNA sequences to capture the target RNA–protein complexes. The advantage of these mass spectrometry-based techniques is to be able to capture RNA–protein interactions under natural conditions. However, it is difficult to design DNA fragments suitable for such experiments. Therefore, the desire for a widely applicable detection method for the RNA–protein interaction of specific RNAs that involves *in vivo* labeling without *in vitro* manipulation remains unfulfilled.

Recently, a number of CRISPR-based RNA-targeting Cas nucleases have been reported. These nucleases specifically bind and cleave target RNAs in the presence of guide RNA. This feature, which involves the tracking of target RNAs and the editing of specific bases of RNAs, allows researchers to manipulate specific RNAs (9–11). The proximity-labeling system PUP-IT has been used to study protein-protein interactions (12). With PUP-IT, a proximity ligase PafA derived from bacteria is fused with a bait protein and mediates the ligation of a small protein PupE to lysines on the surrounding proteins. A carboxylase domain containing biotin is tagged at the N-terminus of PupE for streptavidin pulldown of labeled proteins. These are advantageous elements for developing a new tool to capture RNA–protein interactions of specific RNAs. In this report, we describe the CRISPR-based RNA-United Interacting System (CRUIS), a new RNA-centric method for studying RNA-binding proteins on specific RNAs using engineered dCas13a and PUP-IT. 

## MATERIALS AND METHODS

### Cell culture and generation of stable cell line

HEK293T cells were grown in DMEM (Hyclone) supplemented with 10% FBS (Biological Industries) in a humidified incubator at 37°C with 5% CO_2_. All constructs were prepared using E.Z.N.A.^®^ Endo-free Plasmid DNA Mini Kit (Omega, cat. #D6950-01B) and transfected with Lipofectamine 2000 (Thermo, cat. #11668019). The sequence of CRUIS is available in the supplementary information. Stable cell lines were generated with the piggyBac transposon system, which is widely applicable to various cell lines including non-mammalian cell lines. GFP-positive cells were enriched by flow sorting after transfection. Single colonies were picked, expanded, and tested via PCR, western blot, and enzyme activity identification for PafA. The HEK293T cell line with the best inducibility (referred to as 293T-CRUIS) was expanded and used for all subsequent experiments.

### Plasmid construction

The CRUIS construct (*dLwaCas13a–PafA-P2A-EGFP*) was generated by subcloning *dLwaCas13a* fused with *PafA* at the C-terminus and a self-cleaving P2A peptide-linked EGFP (enhanced green fluorescent protein) into a *piggyBac* transposon backbone. dLwaCas13a was obtained by introducing two point mutations (R474A and R1046A) in the LwaCas13a (Addgene plasmid #90097) HEPN domains. The *PafA* was obtained from *pEF6a-CD28-PafA* (Addgene plasmid #113400). ClonExpress MultiS One Step Cloning Kit (Vazyme, cat. #C113-01) and Mut Express II Fast Mutagenesis Kit V2 (Vazyme, cat. #C214-01) were used for construct generation. The CRUIS plasmid has been deposited to the open-access platform Addgene.

### Tracking stress granules by CRUIS

293T-CRUIS cells were plated in 24-well tissue culture plates on poly-d-lysine coverslips and transfected with 500 ng *ACTB*-sgRNA, and then 100 mM sodium malonate was applied for 1.5 h before fixing and permeabilizing the cells. For immunofluorescence of G3BP1, cells were blocked with 5% BSA and incubated overnight at 4°C with anti-G3BP1 primary antibody (Proteintech, cat. # 13057-2-AP), and anti-myc primary antibody (Cell Signaling, cat. # 9B11). Cells were then incubated for 2 h at room temperature with secondary antibody and mounted using the anti-fade mounting medium.

### RNA extraction and quantitative real-time PCR

Total RNAs from 5 × 10^5^ 293T cells were extracted with Trizol (Invitrogen, Cat. # 15596026) and RNA concentration were determined by NanoDrop 2000c (Thermo Fisher). cDNA was synthesized using 1 μg RNA by the reverse transcription kit PrimeScript™ II 1st Strand cDNA Synthesis Kit (TaKaRa, Cat. # 6210A) according to the manufacturer's instructions. Each qRT-PCR reaction was performed with cDNA transcribed from 25 ng RNA in a final volume of 20 μl with ChamQ™ SYBR Color qPCR Master Mix (Vazyme Cat. # Q431-03), assayed by QuantStudio™ 7 Flex (Life Technologies). The qPCR data were normalized to GAPDH expressions by relative quantification (ΔΔCt) method. The primers used were: CXCR4 (forward primer, 5′-ACTACACCGAGGAAATGGGCT-3′; reverse primer, 5′-CCCACAATGCCAGTTAAGAAGA-3′), p21 (forward primer, 5′-TGTCCGTCAGAACCCATGC-3′; reverse primer, 5′-AAAGTCGAAGTTCCATCGCTC-3′); NORAD (forward primer, 5′-CAGAGGAGGTATGCAGGGAG-3′; reverse primer, 5′-GGATGTCTAGCTCCAAGGGG-3′), β-actin (forward primer, 5′-CATGTACGTTGCTATCCAGGC-3′; reverse primer, 5′-CTCCTTAATGTCACGCACGAT-3′). GAPDH (forward primer, 5′-AGATCCCTCCAAAATCAAGTGG-3′; reverse primer, 5′-GGCAGAGATGATGACCCTTTT-3′).

### Western blot

293T-CRUIS cell lines transfected with or without pCMV-bio-pupE were analyzed by western blot. About 2 million cells were harvested and washed with cold PBS. Lysis buffer (1% Triton, 50 mM Tris 7.5, 150 mM NaCl) with 100× protease inhibitor was added to the pellet. Cells were resuspended and incubated on ice for 1 h. Then the lysate was spun down and the supernatant collected with the addition of protein loading buffer. The samples were boiled at 100°C for 10 min and loaded on 4–20% SDS-PAGE gels, followed by immune-bolting with anti-myc antibody and streptavidin-HRP (Cell Signaling, cat. # 3999s) to identify the expression of dCas13a-PafA fusion protein and the activity of PafA ligase.

For the enrichment of Bio-PupE modified proteins by streptavidin magnetic beads. Thirty-six hours after transfection with sgRNA or non-target sgRNA into the 293T-CRUIS cell line, the treated cells were harvested and lysed using cell lysate buffer. 20 μl streptavidin magnetic beads used for capturing labeled proteins from cell lysate supernatant and washed three times by wash buffer (8 M urea, 50 mM Tris 8.0, 200 mM NaCl). The obtained proteins were boiled at 100°C for 20 min and used for western blot to analyze whether HNRNPK was modified by Bio-PupE, HNRNPK was identified by specific antibody (Proteintech, cat. #11426-1-AP).

### Mass spectrometry preparation

About 30 million cells transfected with pCMV-bio-pupE and sgRNA were used for the mass spectrum. Cells were harvested and washed with cold PBS, then incubated with 2 ml lysis buffer at 4°C. After shaking for 1 h, the lysate was spun down at 4°C for 10 min. The supernatant was transferred into new tubes, with the addition of urea and DTT to a final concentration of 8 M and 10 mM. The lysate was incubated at 56°C for 1 h, then treated with 25 mM iodoacetamide in the dark for 45 min to aminocarbonyl modify the Cys site of proteins. 25 mM DTT was added to terminate the modification. Streptavidin–biotin magnetic beads were washed with 500 μl PBS three times and then resuspended in lysis buffer with an equal volume of beads. The lysate was then added 50 μl beads and it was incubated on a rotator at 4°C overnight. The beads were washed with the following buffers: twice with buffer 1 (50 mM Tris 8.0, 8 M urea, 200 mM NaCl, 0.2% SDS), once with buffer 2 (50 mM Tris 8.0, 200 mM NaCl, 8 M urea), twice with buffer 3 (50 mM Tris 8.0, 0.5 mM EDTA, 1 mM DTT), three times with buffer 4 (100 mM ammonium carboxylate), and finally the beads were resuspended in 100 μl buffer 4. Trypsin, 4 μg (Promega, cat. # v5113) was added to digest overnight at 37°C. The peptides were collected with ziptip by the addition of 1% formic acid, then washed with 0.1% TFA (Sigmal, cat. # 14264) and eluted in 50 μl of 70% ACN (Merck Chemicals, cat. # 100030) –0.1% TFA. The peptides were analyzed on an Orbitrap Fusion.

### Mass spectrometry data analysis

For statistical analysis, the R package Limma (13) was applied for the analysis of LFQ intensity data as previously reported (14–16). The target RNA binding proteins were determined by a moderated *t*-test (*P*-value < 0.05) and fold change (fold change > 3). Previously reported RNA binding proteins were obtained from StarBase v2.0 (http://starbase.sysu.edu.cn/) (17). The R package clusterProfiler was used to identify significantly enriched biological processes in the RNA interactome (*P*-value cutoff = 0.01, *q*-value cutoff = 0.05, *p*.adjust method = Benjamini & Hochberg). The subcellular localization of the identified RBPs was analyzed by an online gene annotation & analysis resource ‘Metascape’ (www.metascape.org). All data visualization was implemented in R using the ggplot2 package.

### RNA immunoprecipitation

For RNA immunoprecipitation experiments, HEK293T cells were plated in a 6-cm dish and transfected with target protein expression plasmid (labeled with HA-tag at the C-terminus). Thirty-six hours after transfection, proteins were crosslinked to RNA by adding formaldehyde drop-wise directly to the medium to a final concentration of 0.75% and rotating gently at room temperature for 15 min. After crosslinking, 125 mM glycine in PBS was used for quenching, and the cells were incubated for 10 min at room temperature. Cells were washed with ice-cold PBS, harvested by scraping, and the cell suspension was centrifuged at 800 g for 4 min to pellet the cells. Cells were lysed with RIPA buffer supplemented with Protease Inhibitor Cocktail, EDTA-free and Recombinant RNasin^®^ Ribonuclease Inhibitor (Promega cat. # N2515). Cells were allowed to lyse on a rotator for 20 min at 4°C and then sonicated for 2 min with a 30 s on/30 s off cycle at low intensity on a Bioruptor sonicator (Diagenode) at 4°C. Insoluble material was pelleted by centrifugation at 16 000g for 10 min at 4°C, and the supernatant containing the clarified lysate was split into two portions for pulling down with anti-HA magnetic beads (bimake cat. # B26202) or mouse IgG-conjugated magnetic beads overnight in a rotator at 4°C. After incubation with sample lysate, beads were pelleted, washed three times with RIPA buffer, and then washed with 1× DNase buffer (RNase-free). Beads were resuspended in 100μl DNase buffer (RNase-free). DNase I (RNase-free) was added, followed by incubation at 37°C for 30 min on a rotator. Proteins were then digested by the addition of Proteinase K (Takara cat. # 9034) for about 2 h at 37°C with rotation. After that, MicroElute RNA Clean Up Kit (Omega cat. # R6247-01) was used for RNA purification. Purified RNA was reverse transcribed to cDNA using PrimeScript™ II 1st Strand cDNA Synthesis Kit (TaKaRa, cat. # 6210A), and pulldown was quantified with qPCR using ChamQ™ SYBR Color qPCR Master Mix (Vazyme cat. # Q431-03) and the Life Technologies QuantStudio™ 7 Flex. Enrichment was quantified for samples compared with their matched IgG antibody controls. The primers used for RIP-qPCR were: forward primer, 5′-GACAGGCCGAGCCCTCTGC-3′; reverse primer, 5′-GGCTTCAAGGTCTGGGCACAGC-3′.

## RESULTS AND DISCUSSION

### Strategies for developing CRUIS

Human cells encode a large number of RNAs, including many non-coding RNAs. These RNAs are expressed differentially in various cells and physiological conditions. However, the functions and regulatory mechanisms of the majority of these transcripts remain unknown. One potential key to understanding is the RNA-binding protein, which is a feature throughout the entire life cycle of RNA (including mRNA, lncRNA, etc.), indicating the importance of the study of detailed RNA–protein interactions.

We used the CRISPR-based RNA-target Cas nuclease as an RNA tracker to bring the proximity-labeling system to the designated target RNA (12,18). CRUIS can capture RNA–protein interactions of specific RNA sequences effectively. In CRUIS, dead RNA-guided RNA targeting nuclease LwaCas13a (dLwaCas13a) (9) is used as a tracker to target specific RNA sequences, while the proximity labeling enzyme PafA is fused to dLwaCas13a to label any surrounding RNA-binding proteins. Subsequently, the labeled proteins are enriched and identified by mass spectrometry (Supplementary Figure S1).

**Figure 1.**
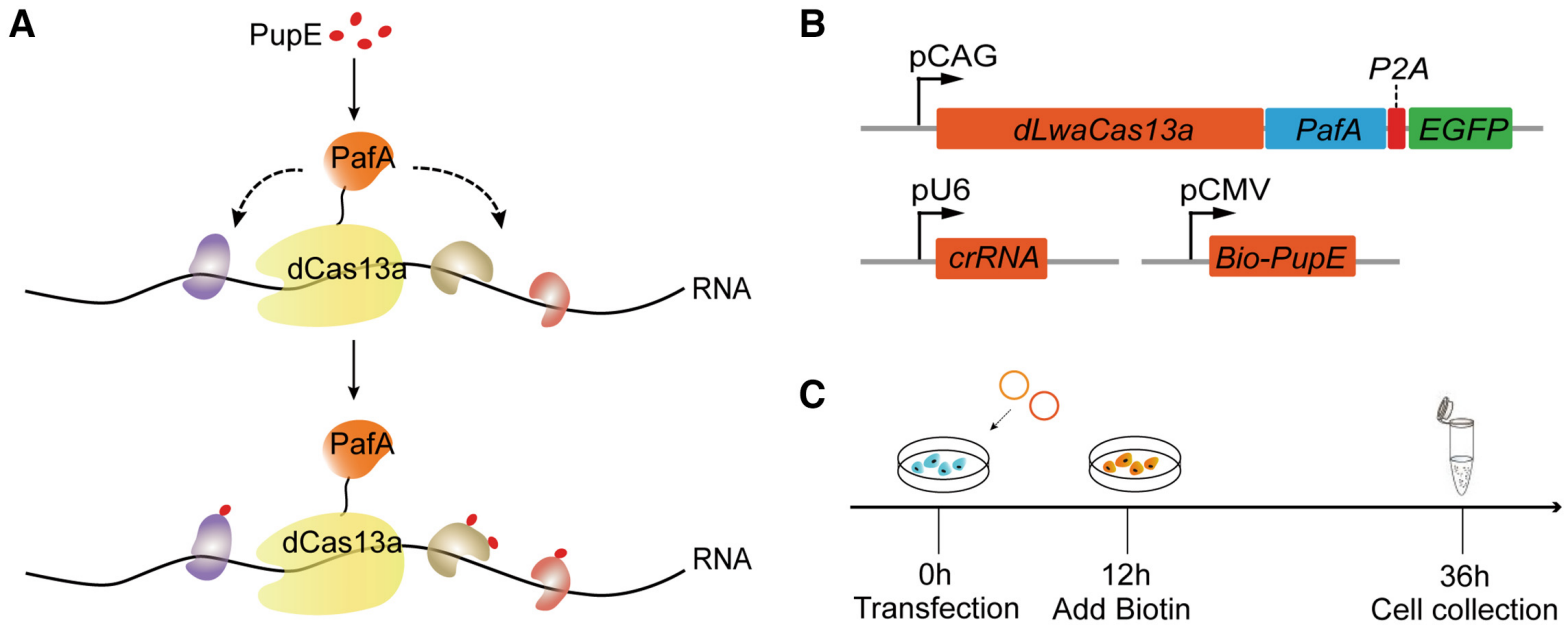
Design of CRUIS. (**A**) Schematic of the CRISPR-based RNA targeting, proximity targeting system. PafA is fused to dLwaCas13a protein and mediates PupE modification of the surrounding proteins of the target RNA. (**B**) Plasmids involved in CRUIS. (**C**) Timeline for CRUIS to capture RNA–protein interaction.

Using this strategy, proteins that interact with specific RNAs can be labeled in living cells, which avoids the risk of RNA degradation introduced by processing RNA–protein complexes in vitro. In addition, this method avoids over-expressing the target RNA with the MS2-tag sequence in the cell, so the abundance of the target RNA in the cell is in a natural state and the acquired RNA represents the real situation. In short, we capture RNA–protein interactions of specific RNAs via Pup ligase PafA fused to the dLwaCas13a and targeting specific RNA loci with single guide RNAs (sgRNAs) (Figure 1A).

### Constructing CRUIS

In comparison to the existing methods, CRUIS shows quite a few advantages. First, it provides a simple and effective way to obtain potential RNA-binding proteins of target RNA. Second, CRUIS can identify RNA–protein interactions in a natural state. Finally, CRUIS can label potential RNA-binding proteins in living cells, thereby avoiding the manipulation of RNA *in vitro* and decreasing the impact of RNA degradation.

To implement CRUIS in cells, we first constructed a transfection vector which fused *dLwaCas13a* and *PafA*, and then cloned the fused d*LwaCas13a–PafA* gene in-frame with the self-cleaving P2A peptide sequence and *EGFP*, and the fusion gene driven by a CAG promoter (Supplementary Figure S2, Supplementary Table S1). In addition, because PafA is mainly expressed in cytoplasm, in order to enable CRUIS to be widely applied to RNA distributed in the nucleus and cytoplasm, we introduced NLS sequences (Figure 1B, Supplementary Figure S2). Using EGFP we observed that the introduction of NLS does not result in the complete distribution of CRUIS in the nucleus due to PafA, but in the nucleus and cytoplasm, which confers versatility (Supplementary Figure S3). In order to express dLwaCas13a-PafA at certain levels, we created a monoclonal HEK293T cell line with stably integrated *dLwaCas13a-PafA* (referred to as 293T-CRUIS) by the *piggyBac* transposon system. For 293T-CRUIS cells, it is only necessary to transfect an expression vector of sgRNA and PupE to achieve the labeling of the RNA-binding proteins of target RNAs (Figure 1C). The obtained monoclonal cell line was to be used for further testing, including whether the dLwaCas13a-PafA fusion protein had proximity targeting activity and whether it could bind to the target RNA.

### Detecting the proximity targeting activity

**Figure 2.**
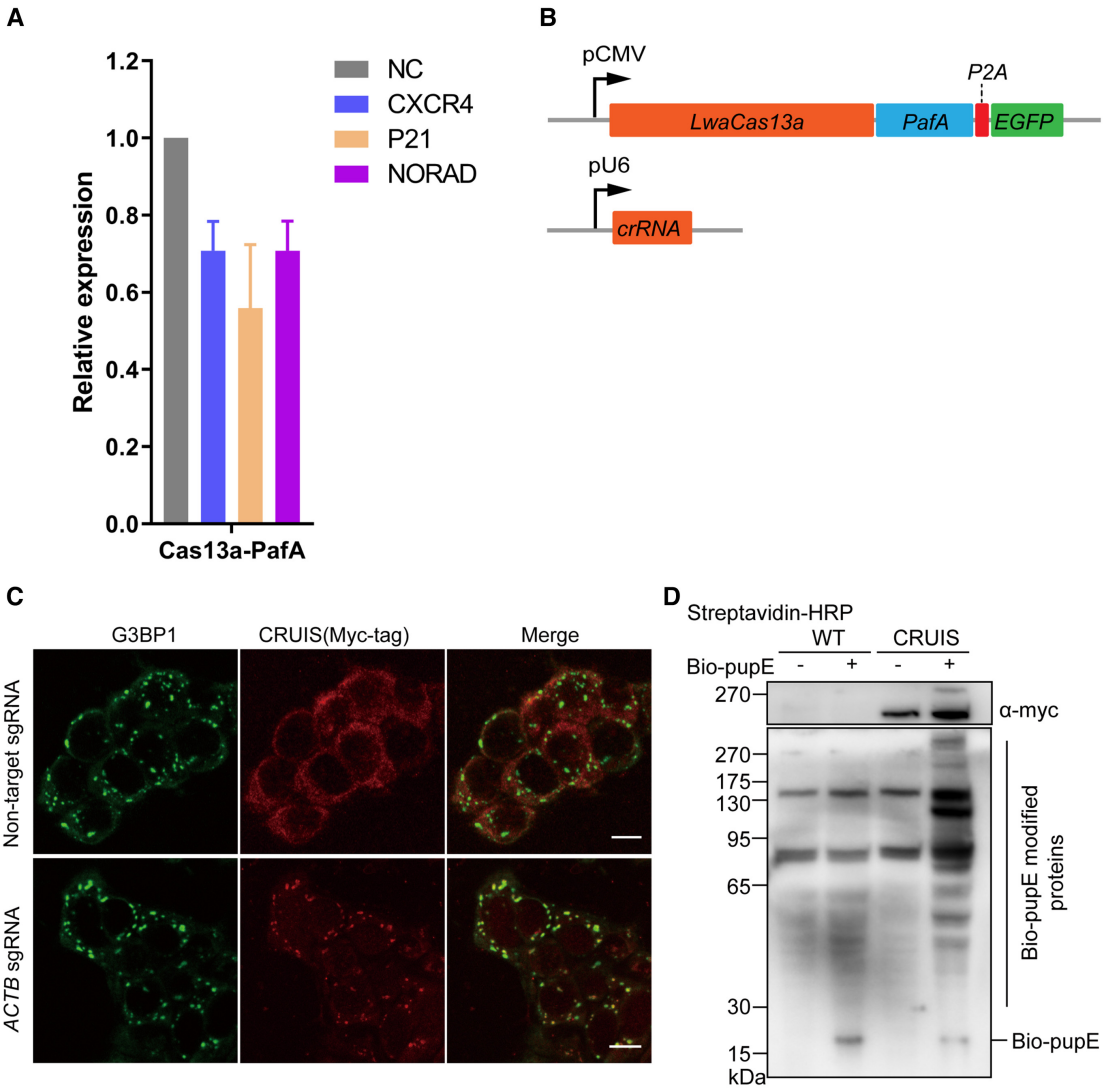
Testing the activity of CRUIS. (**A**) HEK239T cells were co-transfected with LwaCas13a-PafA and sgRNA expression plasmid to detect the mRNA expression level of the target gene after 24 hours; non-target sgRNA was used as the negative control (*n* = 3, mean ± S.E.M). (**B**) Plasmids used in this assay. (**C**), Representative immunofluorescence images of 293T-CRUIS cells treated with 100 mM sodium malonate (scale bar 10μm). Stress granules are indicated by G3BP1 staining. (**D**) Testing the proximity label activity of CRUIS.

To determine whether CRUIS can bind to the target RNA, retain normal catalytic activity, and label surrounding proteins, we first selected several 293T-CRUIS cell lines and determined the proximity targeting activity. We confirmed that PafA retained the ability to label adjacent proteins in 293T-CRUIS cells (Supplementary Figure S4). In addition, we investigated whether CRUIS could bind to the target RNA. Since binding to the target RNA is a prerequisite for clearance, we first examined whether LwaCas13a-PafA could knock down the expression level of the target RNA. As expected, LwaCas13a-PafA performed well in knocking down target RNA (Figure 2A and B, Supplementary Figure S5, Supplementary Table S2).

To further confirm whether CRUIS would be able to recognize target RNA with a specific sgRNA, we used *ACTB*-targeted sgRNA to determine whether CRUIS colocalizes with ACTB-containing stress granules under conditions induced by sodium malonate (9,19,20). Twenty-four hours after transfecting *ACTB*-targeting sgRNA into the 293T-CRUIS cell line, stress granules were induced by adding 100 mM sodium malonate into the culture medium. Immunochemical labeling with an antibody against the stress granule marker G3BP1 demonstrated that CRUIS had been recruited specifically into the stress granules (Figure 2C).

### Capturing RBPs of *NORAD*

To prove the concept, we applied CRUIS to study the RBPs of *NORAD*, a long non-coding RNA. *NORAD* plays an important role in genomic stability. Moreover, previous studies have suggested that RBPs are critical for the function of *NORAD* (16). To this end, we transfected the *NORAD*-target sgRNA into the 293T-CRUIS. Biotin was added to the medium at 12 hours after the transfection. Twenty-four hours later, the cells were collected and lysed (Figure 1C) Then, we pulled down all biotinylated proteins using streptavidin beads. Finally, LC–MS/MS was used to identify the proteins enriched by affinity-based purification (Supplementary Figure S1).

**Figure 3.**
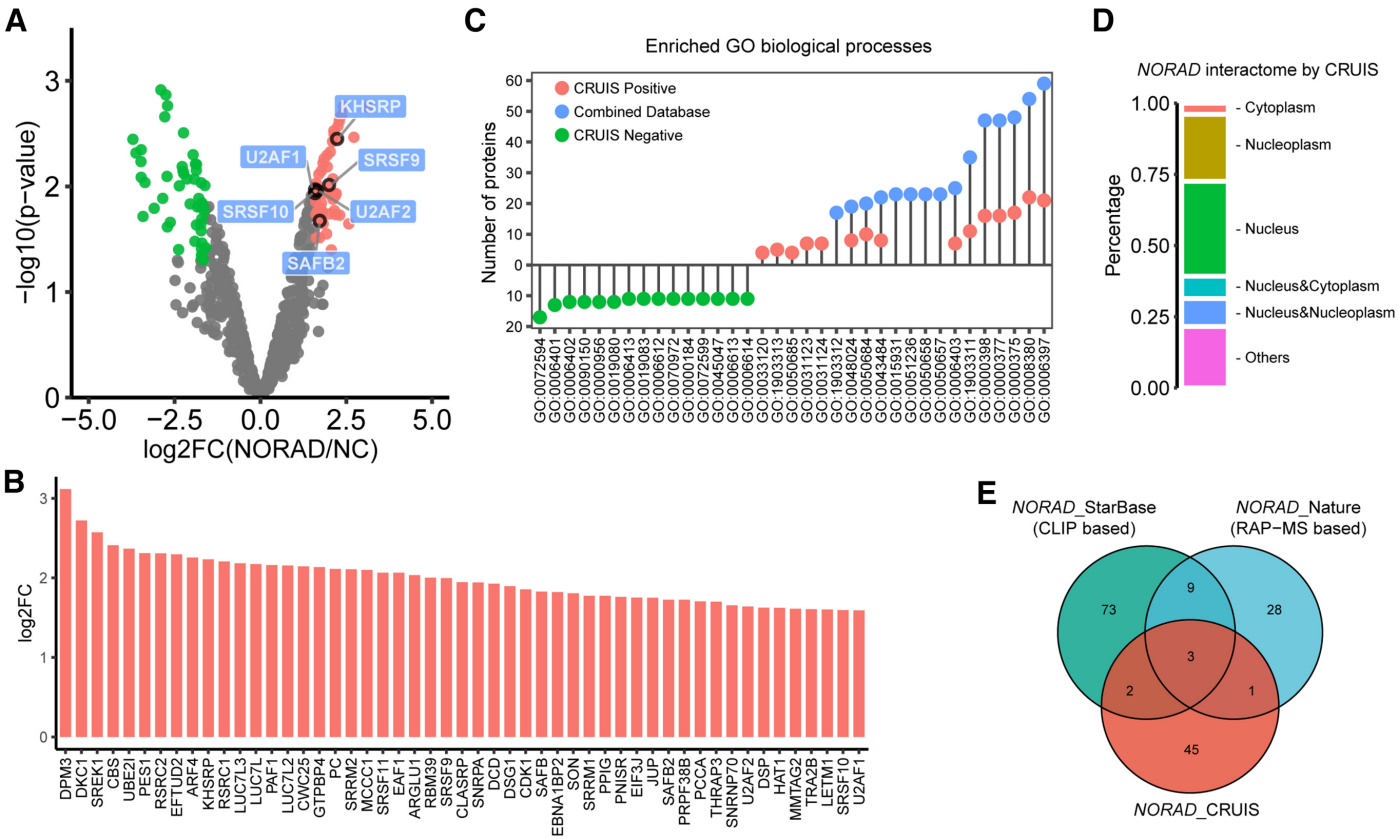
Capturing RNA-binding proteins of *NORAD* by CRUIS. (**A**) The target RBPs were determined by a moderated t-test (*P* value < 0.05) and fold change (fold change > 3). (**B**) Bar plot of log2 fold change (log_2_FC) of the identified proteins in NORAD interactome by CRIUS. (**C**) The top 15 GO-enriched biological processes of proteins in *NORAD* interactome by CRUIS (red dots), the negative control (green dots) and combined datasets (light blue dots). (*P*-value < 0.01, *P*.adjust < 0.05). (**D**) Subcellular distribution of the identified proteins in *NORAD* interactome by CRIUS. (**E**) Comparison of *NORAD* interactome by CRUIS with the two public datasets: RAP MS (16) and StarBase v2.0 database (17).

We found that 51 candidates were significantly enriched in the *NORAD* targeting sgRNA group (*P*-value < 0.05) compared with the non-targeting sgRNA control group (Figure 3A). Among those 51 candidate proteins, six (KHSRP, SRSF9, U2AF2, SRSF10, U2UF1 and SAFB2) are previously reported *NORAD* binding proteins (16,17). The enrichment of each protein, reflected by the fold changes, is also ranked (Figure 3B). The top hits include DKC1, SREK1 and RSRC2, which are known RNA binding proteins that play important roles in regulating RNA splicing and mRNA processing (1,17).

The candidate *NORAD*-binding proteins identified by CRUIS are involved in biological processes that are distinct from those of the control sample (Figure 3C, Supplementary Table S3). The top biological processes characterized as related to the function of *NORAD* binding proteins are RNA splicing (GO:0008380), mRNA processing (GO:0006397), and RNA splicing via transesterification reactions (GO:0000375). Furthermore, the subcellular localization analysis of the identified *NORAD*-binding proteins also shows a significant enrichment of nuclear proteins (Figure 3D).

**Figure 4.**
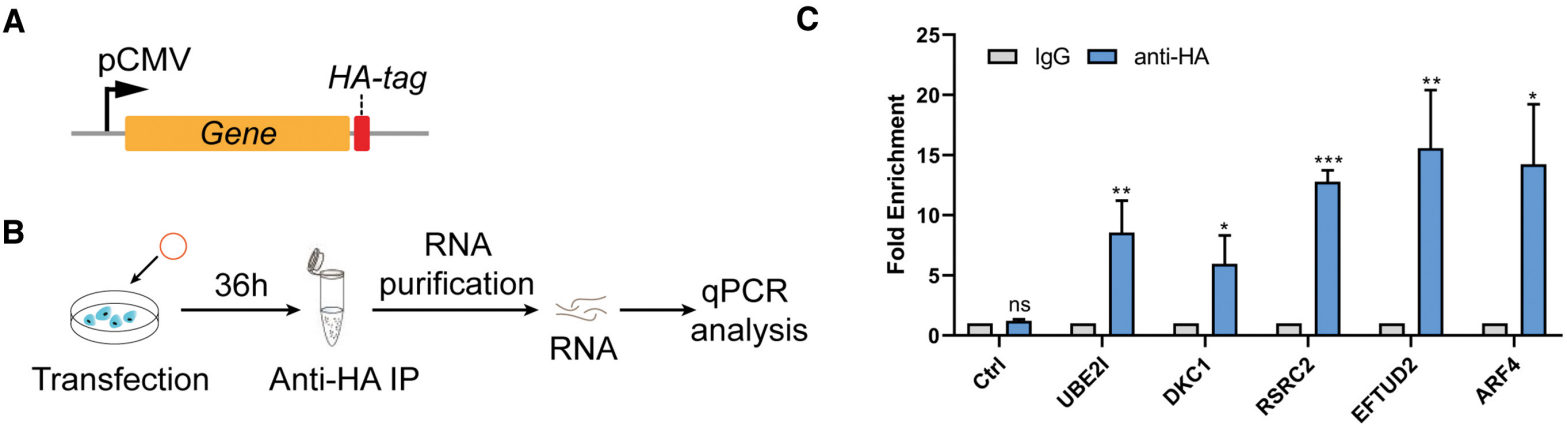
Validation of proteins enriched by RIP-qPCR. (**A**) The pattern diagram shows that the marker protein is HA-tag at the C-terminus for subsequent RIP. (**B**) Schematic of RNA immunoprecipitation for quantification of RNA–protein interaction. (**C**) Some proteins found by CRUIS could significantly enrich NORAD transcript compared with the anti-IgG group and control (n = 3, mean ± S.E.M. ***P < 0.001; **P < 0.01; *P < 0.05).

Using CRUIS, we verified some *NORAD*-binding proteins identified previously (Figure 3E) (16). Furthermore, we performed RIP-qPCR to confirm several new binding proteins of *NORAD* from the enriched proteins (Figure 4A–C).

### Capturing RBPs of *p21* mRNA

To determine whether CRUIS is able to identify RBPs for mRNAs, we designed sgRNAs to target *p21* mRNA and applied CRUIS. Our data from mass spectrometry retrieved putative RBPs for *p21* mRNA, some of them are known RBPs of p21 mRNA (marked in red) (Supplementary Figure S6A). We verified that CRUIS can mediate Bio-PupE modification on an RNA-binding protein associating with p21 mRNA (Supplementary Figure S6B). The enriched proteins of p21 mRNA are different from the RBPs of NORAD captured by CRUIS. Some of the proteins enriched in the p21-target group, such as HNRNPK, HNRNPA1, HNRNPC and PCBP2, are common proteins that bind most nascent hnRNA. It reflects the different post-transcriptional maturation mechanism between mRNA and long non-coding RNA.

### Advantages of CRUIS

Here we have described a new effective tool for capturing RNA–protein interactions. The proximity targeting system PUP-IT is brought into contact with an RNA by dCas13a to label the surrounding proteins, providing a powerful tool for finding potential RNA-binding proteins of specific RNAs. This RNA-centric RBP-capturing strategy overcomes the limitations of the existing RNA-centric methods for studying RNA–protein interactions.

By using CRUIS, it is not necessary to express target RNAs with tags such as MS2 to facilitate immunoprecipitation or to design a biotin-labeled DNA probe for a specific RNA. In CRUIS-positive cells only sgRNAs are required, and the RBP labeling is completed in living cells without manipulating RNA–protein complexes *in vitro*, which maximally avoids the possible disruption of RBP complexes. And the low off-target characteristics of LwaCas13a provide a guarantee for CRUIS to accurately binding to the target RNA (9).

A previous report described a biotinylated dCas9-based method for simultaneously studying long-range DNA interactions and chromatin-associated proteins by binding to a specific locus with FB (flag and biotin-acceptor-site)-tagged dCas9 protein and introduced BirA, which marks FB-tagged dCas9 by biotin for subsequent purification and sequencing (21). In addition, a new tool named GLoPro which using dCas9 infusion with APEX2 has been shown to be useful for capturing DNA–protein interactions, with many advantages over traditional methods for studying DNA-protein interactions such as ChIP (chromatin immunoprecipitation assay) (22). In contrast, the GLoPro directly labels proteins surrounding specific gene loci in living cells.

Importantly, our results also show that CRUIS can be universally used for different types of RNA, including lncRNA and mRNA. This characteristic is dependent on dCas13a, which does not show selectivity for the type of RNA. This indicates that CRUIS has broad applicability potentials.

### Limitations of CRUIS

Since the guide RNA activity remains difficult to predict and the secondary structure of RNAs remains incomplete, we recommend testing multiple guide RNAs to select potent guides for analysis. Given the differences in length of RNA, theoretically, one sgRNA does not represent the intact pattern of a whole RNA. Nevertheless, CRUIS provides a potential tool for studying RNA-binding proteins at specific positions on target RNAs. For the working window of CRUIS, without considering the secondary structure of RNA, the labeling radius is controlled by the linker length between dCas13a and PafA. In the case of CRUIS, we used a 19-aa linker linked to the C terminus of dCas13a, ∼7 nm. Considering the size of Cas13a, we estimate the labeling radius of dCas13a-PafA is 17 nm, covering about 50 bases if in a stretched conformation.

CRUIS still faces some challenges as the spatial structural uncertainty of the target RNA and the efficiency of sgRNAs. Understanding the secondary structure of the target RNA is useful for the effective implementation of CRUIS. The efficiency of sgRNA is an important factor, therefore, efficient sgRNA must be selected before applying CRUIS. Because the CRISPR complex is large, CRISPR-based targeting might affect the structure of the targeted RNA, which in turn changes the patterns of interacting proteins.

### Perspectives

CRUIS is a hybrid of two molecular machineries, CRISPR-based RNA-targeting Cas nucleases (dCas13a) and a proximity-labeling system (PUP-IT). Our results demonstrate that CRUIS is feasible and specific for the identification of RBPs of both non-coding RNAs and mRNAs. The universality of dCas13a and PUP-IT in mammalian cells promises a broad application prospect. Individual sgRNAs can be used to guide CRUIS to label the RBPs at a certain position on a target RNA. Therefore, CRUIS can potentially ‘cruise’ along the target RNA molecule to generate the RNP atlas of an RNA.

## Supplementary Material

gkaa143_Supplemental_File
