# Supplementary material for: Capturing RNA–protein interaction via CRUIS
Source: Nucleic Acids Res. 2020 Mar 6;48(9):e52. doi: 10.1093/nar/gkaa143 (PMC7229851; doi:10.1093/nar/gkaa143)
Supplement: gkaa143_Supplemental_File [file gkaa143_supplemental_file.pdf]

Capturing RNA-protein interaction via CRUIS

Ziheng Zhang[1,2,3,#], Weiping Sun[1,2,3,#], Tiezhu Shi[1,2,3,#], Pengfei Lu[1,*], Min Zhuang[1,*] & Ji-Long Liu[1,*]

SUPPLEMENTARY INFORMATION

Supplementary figures S1-S6

Supplementary tables S1-S3

Supplementary figures S1-S6

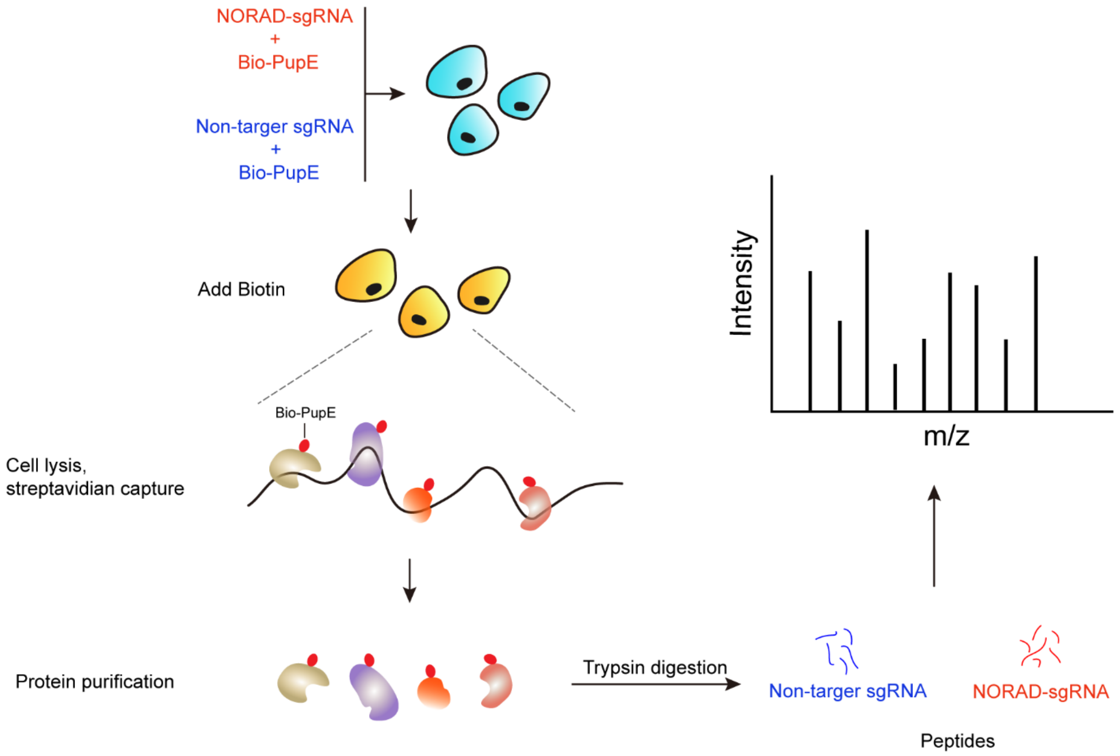

Supplemental figures S1. A workflow of CRUIS to identify the RNA-protein interactions.

Cells were cultured in 150 mm dishes; 12 hours after transfection (sgRNA and pCMV-Bio-PupE) biotin was added to make the final conc. 20 μM; 24 hours after addition of biotin the cells were collected and lysed. Streptavidin-beads were used for enriching and purifying proteins labeled with Bio-PupE. Finally, the type and abundance of proteins were identified by protein mass spectrometry after digestion by trypsin.

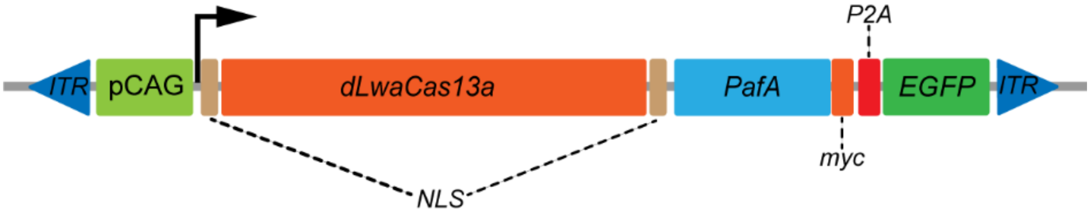

Supplemental figures S2. A diagram of the CRUIS plasmid.
NLS, nuclear localization sequence; pCAG, CAG promoter; *myc*, *myc* epitope tag; P2A, P2A self-cleaving peptide; EGFP, enhanced green fluorescent protein; ITRs, inverted terminal repeats. Thus, the fusion gene is currently too large for viral transduction. We obtained cell lines with stable expression of CRUIS using the piggyBac transposon system. Although the transfection efficiency was low, the GFP-positive cells were enriched by sorting. Single colonies were picked, expanded and tested.

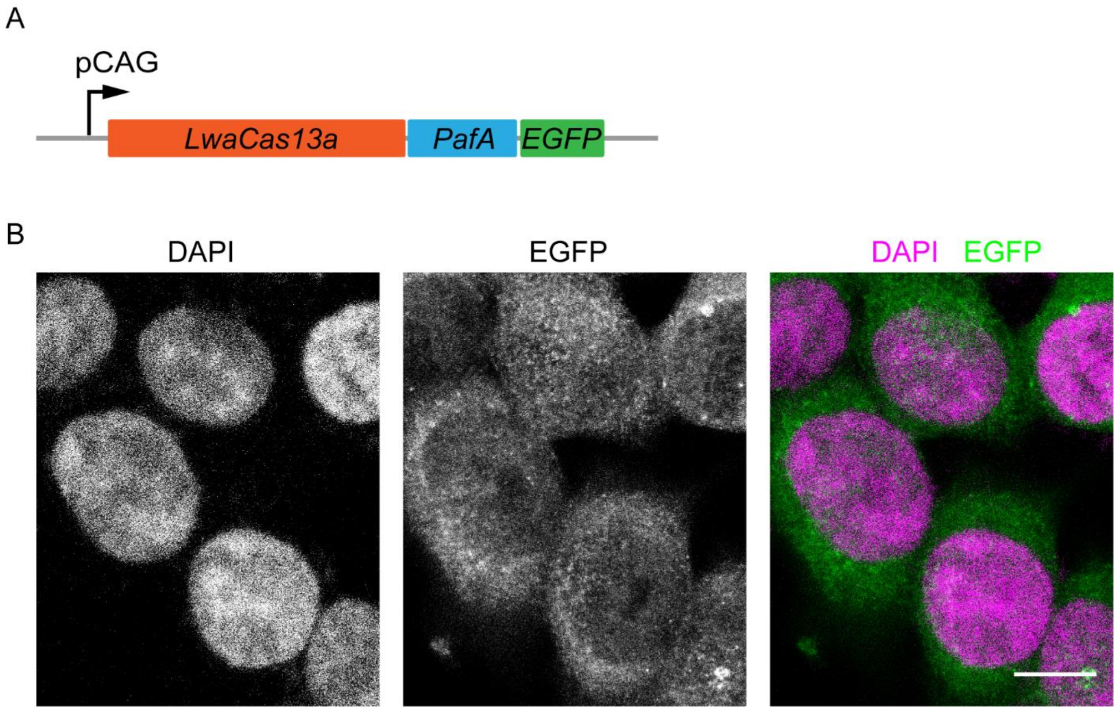

Supplemental figures S3. Subcellular localization of CRUIS.
(A) Schematic diagram of the plasmid structure used in this assay, EGFP was used to label CRUIS in the C-terminus (no *P2A* between *CRUIS* and *EGFP* in the construct). (B) After transfected *pCAG-CRUIS-EGFP* for 24h, the location of CRUIS was determined by EGFP. The results showed distribution in the nucleus and cytoplasm (scale bar 10 μm).

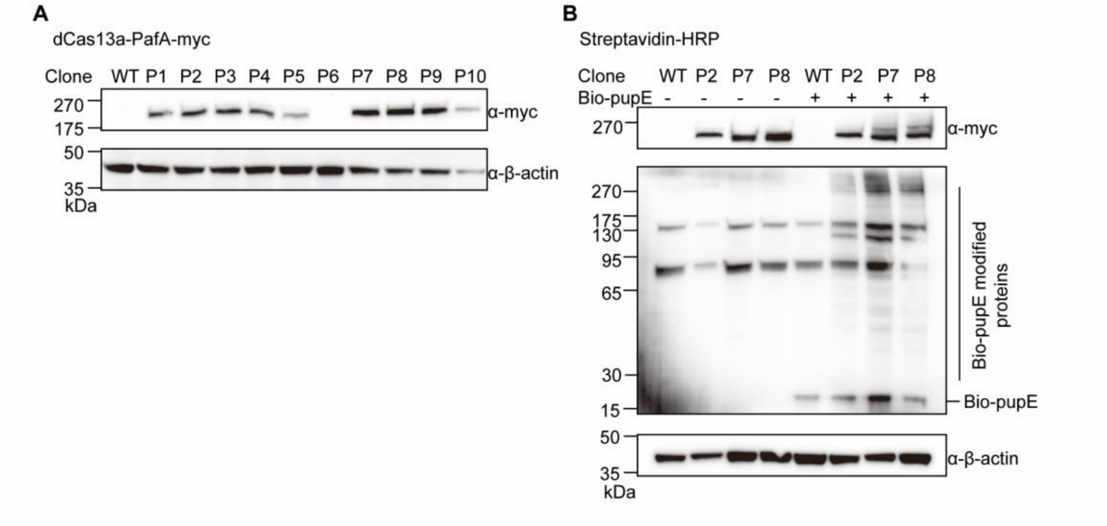

Supplemental figures S4. Selection of CRUIS stable cell lines.

(A) Anti-myc western blotting shows 10 clones with stable expression of CRUIS. (B) Three CRUIS stable cell lines, P2, P7, and P8, were selected to test the enzyme activity of PafA in CRUIS. Anti-streptavidin western blotting indicates that CRUIS shows reliable proximity targeting activity.

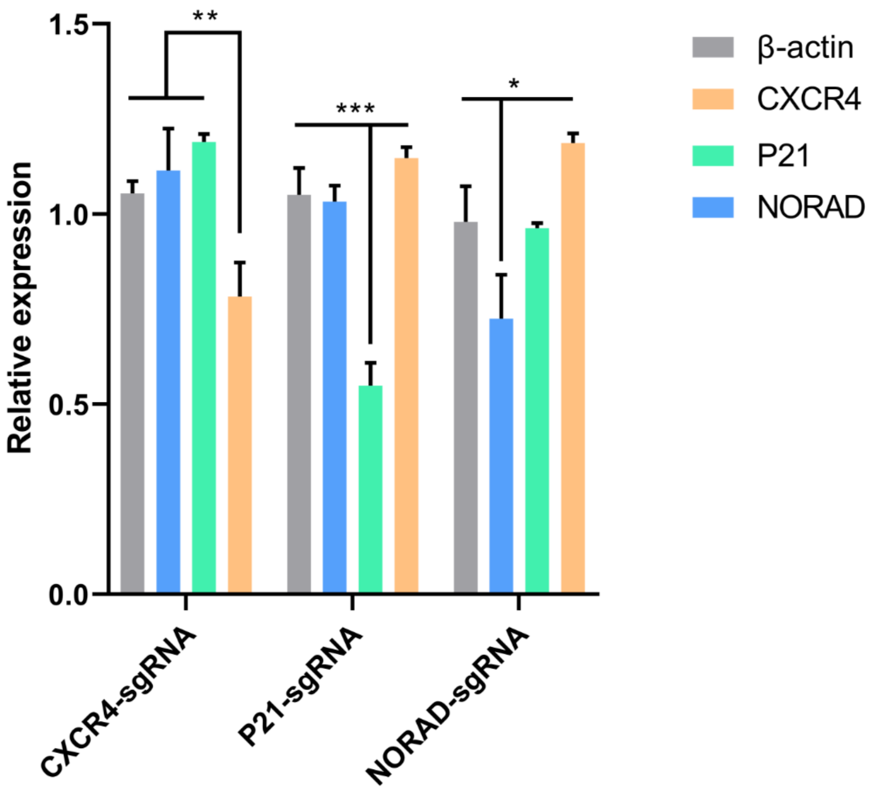

Supplemental figures S5. Expression levels of RNAs. HEK239T cells are co-transfected with LwaCas13a-PafA and sgRNA expression plasmid to detect the mRNA expression level of the target gene after 24 hours. The resulting values were normalized to GAPDH expressions. (n=3, mean ± S.E.M * * * P < 0.001; * * P < 0.01; * P < 0.05).

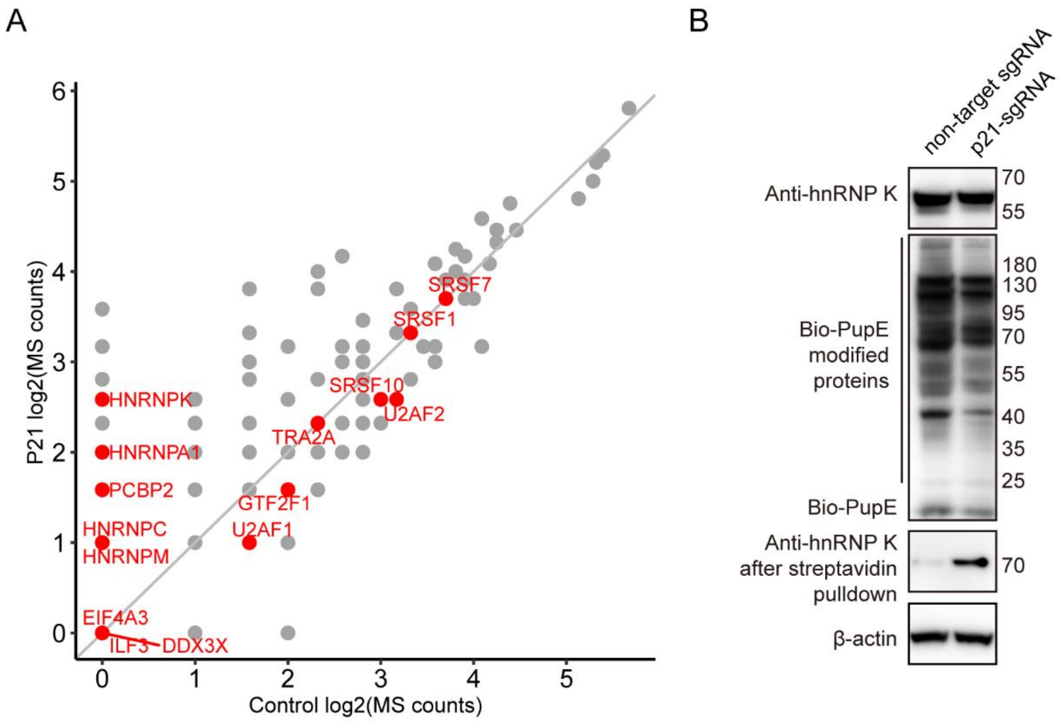

Supplemental figures S6. Obtaining the RNA-binding proteins of P21 mRNA by CRUIS.

(A) RNA-binding proteins of *P21* mRNA were captured by CRUIS. Some proteins were enriched in the P21 group (p21-target sgRNA) compared with control (non-target sgRNA). Some of these were *p21*-binding proteins identified previously (marked in red)(17). The red dots in the scatterplot are examples of known P21 RNA-binding proteins in StarBase v2.0 database. (B) Western blot showed CRUIS-mediated Bio-PupE modification of HNRNPK. After capturing the RBPs of *p21* mRNA by CRUIS, the labeled proteins ware enriched using streptavidin magnetic beads, and HNRNPK was detected by HNRNPK-specific antibody. Compared to the non-target sgRNA group, the p21-target group showed highly enriched of HNRNPK.

Supplementary Table S1. Amino acid sequence of dLwaCas13a-PafA-P2A-EGFP fusion protein.

Name	Amino acid sequence
dLwaCas13a-PafA-P2A-EGFP	MPKKKRKVGRVCRISSLRYRGPGIATMKVTKVDGISHKKYIEEGKLVKSTSEENRT<br>SERLSELLSIRLDIYIKNPDNASEEENRIRRENLKKFFSNKVLHLKDSVLYLKNRKEK<br>NAVQDKNYSEEDISEYDLKNKNSFSVLKKILLNEDVNSEELEIFRKDVEAKLNKINSL<br>KYSFEENKANYQKINENNVEKVGGKSKRNIIYDYYRESAKRNDYINNVQEAFDKLY<br>KKEDIEKLFFLIENSKKHEKYKIREYYHKIIGRKNDKENFAKIIYEEIQNVNNIKELIEKI<br>PDMSELKKSQVFYKYYLDKEELNDKNIKYAFCHFVEIEMSQLLKNYVYKRLSNISN<br>DKIKRIFEYQNLKKLIENKLLNKLDTYVRNCGKYNYYLQVGEIATSDFIARNRQNEAF<br>LRNIIGVSSVAYFSLRNILETENENDITGRMRGKTVKNNKGEEKYVSGEVDKIYNEN<br>KQNEVKENLKMFYSYDFNMDNKNEIEDFFANIDEAISSIAHGIVHFNLELEGKDIFAF<br>KNIAPSEISKKMFQNEINEKKLKLKIFKQLNSANVFNYYEKDVIIKYLKNTKFNFVNK<br>NIPFVPSFTKLYNKIEDLRNTLKFFWSVPKDKEEKDAQIYLLKNIYYGEFLNKFVKNS<br>KVFFKITNEVIKINKQRNQKTGHYKYQKFENIEKTVPVEYLAIIQSREMINNQDKEEK<br>NTYIDFIQQIFLKGFIDYLNKNNLKYIESNNNNDNNDIFSKIKIKKDNKEKYDKILKNYE<br>KHNRNKEIPHEINEFVREIKLGKILKYTENLNMFYLILKLLNHKELTNLKGSLEKYQS<br>ANKEETFSDELELINLLNLDNNRVTEDFELEANEIGKFLDFNENKIKDRKELKKFDT<br>NKIYFDGENIIKHRAFYNIKKYGMLNLLEKIADKAKYKISLKELKEYSNKKNEIEKNYT<br>MQQNLHRKYARPKKDEKFNDEDYKEYEKAIGNIQKYTHLKNKVEFNELNLLQGLLL<br>KILHRLVGYTSIWERDLRFRLKGEFPENHYIEEIFNFDNSKNVKYKSGQIVEKYINFY<br>KELYKDNVEKRSIYSDKKVKKLKQEKKDLYIANYIAHFNYIPHAEISLLEVLENLRKLL<br>SYDRKLKNAIMKSIVDILKEYGFVATFKIGADKKIEIQTLESEKIVHLKNLKKKKLMTD<br>RNSEELCELVKVMFEYKALEQRPQGGGGPKKKRKVGGSGMSTVESALTRRIMGI<br>ETEYGLTFVDGDSKKLRPDEIARRMFRPIVEKYSSSNIFIPNGSRLYLDVGSHPEYA<br>TAECDNLTQLINFEKAGDVIADRMAVDAEESLAKEDIAGQVYLFKNNVDSVGNSYG<br>CHENYLVGRSMPLKALGKRLMPFLITRQLICGAGRIHHPNPLDKGESFPLGYCISQ<br>RSDHVWEGVSSATTRSRPIINTRDEPHADSHSYRRLHVIVGDANMAEPSIALKVGS<br>TLLVLEMIEADFGLPSLELANDIASIREISRDATGSTLLSLKDGTTMTALQIQQVVFE<br>HASKWLEQRPEPEFSGTSNTEMARVLDLWGRMLKAIESGDFSEVDTEIDWVIKKK<br>LIDRFIQRGNLGLDDPKLAQVDLTYHDIRPGRGLFSVLQSRGMIKRWTTDEAILAAV<br>DTAPDTTRAHLRGRILKAADTLGVPVTVDWMRHKVNRPEPQSVELGDPFSAVNSE<br>VDQLIEYMTVHAESYRSEQKLISEEDLGSGATNFSLLKQAGDVEENPGPMVSKGE<br>ELFTGVVPILVELDGDVNGHKFSVSGEGEGDATYGKLTLKFICTTGKLPVPWPTLV<br>TTLTYGVQCFSRYPDHMKQHDFFKSAMPEGYVQERTIFFKDDGNYKTRAEVKFE<br>GDTLVNRIELKGIDFKEDGNILGHKLEYNYNSHNVYIMADKQKNGIKVNFKIRHNIED<br>GSVQLADHYQQNTPIGDGPVLLPDNHYLSTQSALSKDPNEKRDHMVLLEFVTAAG<br>ITLGMDELYK*

Supplementary Table S2. sgRNA information.

Name	Guide sequence (5'-3')	Figure
ACTB-sgRNA	ctggcggcgggtgtggacgggcggcgga	Fig.1C
NORAD-sgRNA	tcggcaacctctttccatctagaagggc	Fig. 2A, Fig. 3A. Fig.S5
CXCR4-sgRNA	atgataatgcaatagcaggacaggatga	Fig. 2A, Fig.S5
P21-sgRNA	tacactaagcacttcagtgcctccaggg	Fig. 2A, Fig. S5, Fig.S6

Supplementary Table S3. Information on biological processes.

ID	Description
GO:0006397	mRNA processing
GO:0008380	RNA splicing
GO:0000375	RNA splicing, via transesterification reactions
GO:0000377	RNA splicing, via transesterification reactions with bulged adenosine as nucleophile
GO:0000398	mRNA splicing, via spliceosome
GO:1903311	regulation of mRNA metabolic process
GO:0006403	RNA localization
GO:0050657	nucleic acid transport
GO:0050658	RNA transport
GO:0051236	establishment of RNA localization
GO:0015931	nucleobase-containing compound transport
GO:0043484	regulation of RNA splicing
GO:0050684	regulation of mRNA processing
GO:0048024	regulation of mRNA splicing, via spliceosome
GO:1903312	negative regulation of mRNA metabolic process
GO:0031124	mRNA 3'-end processing
GO:0031123	RNA 3'-end processing
GO:0050685	positive regulation of mRNA processing
GO:1903313	positive regulation of mRNA metabolic process
GO:0033120	positive regulation of RNA splicing
GO:0006614	SRP-dependent cotranslational protein targeting to membrane
GO:0006613	cotranslational protein targeting to membrane
GO:0045047	protein targeting to ER
GO:0072599	establishment of protein localization to endoplasmic reticulum
GO:0000184	nuclear-transcribed mRNA catabolic process, nonsense-mediated decay
GO:0070972	protein localization to endoplasmic reticulum
GO:0006612	protein targeting to membrane
GO:0019083	viral transcription
GO:0006413	translational initiation
GO:0019080	viral gene expression
GO:0000956	nuclear-transcribed mRNA catabolic process
GO:0090150	establishment of protein localization to membrane
GO:0006402	mRNA catabolic process
GO:0006401	RNA catabolic process
GO:0072594	establishment of protein localization to organelle
